# Genetic diversity of *Plasmodium falciparum *isolates from Pahang, Malaysia based on MSP-1 and MSP-2 genes

**DOI:** 10.1186/1756-3305-4-233

**Published:** 2011-12-13

**Authors:** Wahib M Atroosh, Hesham M Al-Mekhlafi, Mohammed AK Mahdy, Riyadh Saif-Ali, Abdulsalam M Al-Mekhlafi, Johari Surin

**Affiliations:** 1 Department of Parasitology, Faculty of Medicine, University of Malaya, Kuala Lumpur 50603, Malaysia; 2 Department of Molecular Medicine, Faculty of Medicine, University of Malaya, Kuala Lumpur 50603, Malaysia; 3 Department of Parasitology, Faculty of Medicine and Health Sciences, Sana'a University, Sana'a, Yemen; 4 Department of Biochemistry, Faculty of Medicine and Health Sciences, Sana'a University, Sana'a, Yemen

## Abstract

**Background:**

Malaria is still a public health problem in Malaysia especially in the interior parts of Peninsular Malaysia and the states of Sabah and Sarawak (East Malaysia). This is the first study on the genetic diversity and genotype multiplicity of *Plasmodium falciparum *in Malaysia.

**Methods:**

Seventy-five *P. falciparum *isolates were genotyped by using nested-PCR of *MSP-1 *(block 2) and *MSP-2 *(block 3).

**Results:**

*MSP-1 *and *MSP-2 *allelic families were identified in 65 blood samples. RO33 was the predominant *MSP-1 *allelic family identified in 80.0% (52/65) of the samples while K1 family had the least frequency. Of the *MSP-2 *allelic families, 3D7 showed higher frequency (76.0%) compared to FC27 (20.0%). The multiplicity of *P. falciparum *infection (MOI) was 1.37 and 1.20 for *MSP-1 *and *MSP-2*, respectively. A total of seven alleles were detected; of which three *MSP-1 *allelic families (RO33, MAD20 and K1) were monomorphic in terms of size while *MSP-2 *alleles were polymorphic (two 3D7 and two FC27). Heterozygosity (H_E_) was 0.57 and 0.55 for *MSP-1 *and *MSP-2*, respectively.

**Conclusions:**

The study showed that the MOI of *P. falciparum *is low, reflected the low intensity of malaria transmission in Pahang, Malaysia; RO33 and 3D7 were the most predominant circulating allelic families. The findings showed that *P. falciparum *has low allelic diversity with a high frequency of alleles. As a result, antimalarial drug efficacy trials based on MSP genotyping should be carefully interpreted.

## Background

Malaria has been a global health problem threatening more than 40% of the world's population, and about 300-500 million cases of malaria infection are reported every year with approximately one million deaths, mostly among children and pregnant women [1]. Among the malarial parasites, *Plasmodium falciparum*, causes the most severe malarial attacks, is responsible for the high morbidity and mortality, frequent antimalarial drug resistance and aborted vaccines trials [2,3].

Genetic diversity determines the intensity of malaria transmission, thus providing baseline data for any antimalarial drug efficacy trial and the possibility of implementing control strategies based on vaccines. Merozoite surface proteins 1 and 2 (*MSP-1 *and *MSP-2*) are widely used to study the allelic diversity and frequency of *P. falciparum *which are most commonly correlated with the level of transmission in the area under study. The two loci have also been introduced as a discriminatory tool to distinguish new from recrudescent infections [4,5].

Despite the tremendous reduction in malaria annual cases achieved by The Malaria Control Programme (from 55,000 in 1990 to 7010 cases by 2009), malaria continues to be a public health problem in Malaysia, especially in rural and remote areas with *P. falciparum*, the most virulent species, accounting for more than one third of the reported malaria cases (Annual Reports, Ministry of Health, Malaysia). Information about the genetic diversity of *P. falciparum *is lacking in Malaysia. This information is necessary for implementing PCR-based antimalarial drug efficacy trials to examine the current drug policy. Genotyping *P. falciparum *in the antimalarial drug efficacy trials prevents unnecessary change of drug policy due to misclassification of re-infection in the follow-up investigation as recrudescence. The discriminatory power of the genotyping method depends on the allelic diversity and frequency of *P. falciparum *in the study area. Thus, the present study was carried out to investigate the genetic diversity of *P. falciparum *isolated from Pahang, Malaysia using merozoite surface protein-1 (*MSP-1*) and merozoite surface protein-2 (*MSP-2*).

## Methods

### Sampling and malaria microscopy

**Figure 1:**
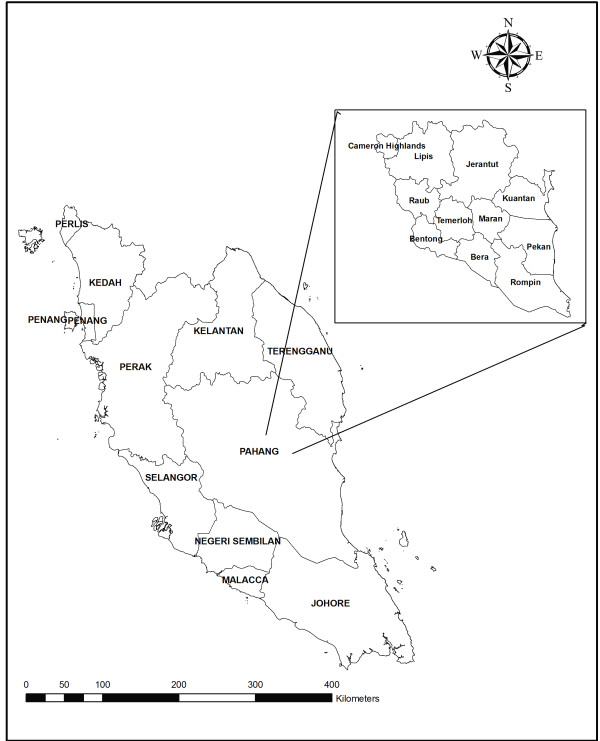
**A geographic map showing Peninsular Malaysia and Pahang's districts**.

A total of 822 blood samples were collected from different districts of Pahang, Malaysia (Figure 1); 728 samples by a survey and 64 by archived positive falciparum malaria slides. In the survey, a finger prick blood sample was taken from each subject for thick and thin blood film and 2-3 drops were collected on 3 MM Whatman^® ^filter paper (Whatman International Ltd., Maidstone, England). Blood films were stained with diluted Giemsa stain and then examined microscopically for the presence of malaria parasites; 200 fields under 1000× magnification were examined from the thick film before the slide was considered negative.

For positive slides, parasite species and stages were reported and parasitaemia (parasite density) was determined by counting only the asexual stages against 300 white blood cells (WBC) and then multiplied by 25; assuming the average of total WBC count of individuals equal to 7500 cells per μl of blood [6]. The level of parasitaemia was graded as low (< 1000 parasites/μl of blood), moderate (1000 - 9999 parasites/μl of blood) and severe (≥ 10,000 parasites/μl of blood). Archived malaria positive slides were also re-examined; parasite species and parasitaemia were recorded. All positive cases of single and mixed falciparum infections were considered for the molecular technique processing.

### Molecular identification and genotyping

Genomic DNA was extracted from blood spots collected on filter papers and from archived Giemsa blood smears. Briefly, a disc of the filter paper was punched out from the blood spot using a pre-flamed paper puncher and placed in 1.5 ml centrifuge tubes using clean, flamed forceps. Regarding archived slides, the slides were first cleaned with chloroform to remove oil. Then, 50 ml of TE buffer was transferred onto the smear and at least half of the smear was completely wiped off the slide using Whatman 1 filter paper and transferred into 1.5 ml microcentrifuge tubes. Genomic DNA was extracted using Qiagen blood and tissue kit (QIAGEN, DNeasy^® ^Blood & Tissue Kit, Cat. no. 69506, Germany) according to the manufacurer's instruction. DNA was eluted using 50 uL AE (10 mM Tris-Cl; 0.5 mM EDTA; pH 9.0) elution buffer (QIAGEN, DNeasy^® ^Blood & Tissue Kit, Cat. no. 69506, Germany) and kept at -20°C until used for PCR. *Plasmodium spp *were identified by 18s rRNA- based nested PCR using genus- and species - specific primers as mentioned previously [7].

*P. falciparum *were further analyzed by amplification of the two highly polymorphic regions of *MSP-1 *(Block 2) and *MSP-2 *(Block 3) using nested-PCR as mentioned previously [8], with slight modifications for the cycling conditions of the secondary PCR. Briefly, oligonucleotide primers sets (Table 1), previously designed by Snounou et al. [8], were used for detecting the different families (K1, MAD20 and RO33 in *MSP-1*; FC27 and 3D7 in *MSP-2*). In the primary PCR, a 50 μl PCR mixture was used containing 5 μl of DNA extract, 1X of MgCl2 free buffer, 1 mM of MgCl_2_, 125 μM of dNTPs, 250 mM of each primer and 1.25 of Taq polymerase enzyme. All reagents were from iNtRON (iNtRON Biotechnology, Inc. Seoul, Korea). Cycling conditions for the primary PCR were as follows; starting with three single steps of denaturation at 94°C for 5 minutes, annealing at 58°C for 2 minutes and extension at 72°C for 2 minutes. This is followed by 25 cycles of denaturation at 94°C for 1 minute, annealing at 58°C for 2 minutes and extension at 72°C for 2 minutes, then a single annealing step at 58°C for 2 minutes and final extension at 72°C for 5 minutes. Three μl of primary PCR product were used as a DNA template in the secondary PCR which had similar concentrations to the primary PCR. The cycling conditions for the secondary PCR were as follows: starting with a single step of denaturation at 95°C for 10 minutes followed by 40 cycles of denaturation at 94°C for 30 seconds, annealing at 58°C for 30 seconds and extension at 72°C for 1 minute, and a final extension at 72°C for 10 minutes. PCR reaction mixtures were incubated in a thermal cycler (MyCycler-BioRad, Hercules, USA).

**Table 1:** Sequences of the primers used to amplify the *MSP-1 *and *MSP-2 *genes of *P. falciparum *isolates from Pahang, Malaysia

Amplification/Gene	Primer	Primer sequence
**Primary PCR**		
**MSP-1**	M1-OF	5^- ^-CTAGAAGCTTTAGAAGATGCAGTATTG-3^- ^
	M1-OR	5^- ^-CTTAAATAGTATTCTAATTCAAGTGGATCA-3^- ^
		
**MSP-2**	M2-OF	5^- ^-ATGAAGGTAATTAAAACATTGTCTATTATA-3^- ^
	M2-OR	5^- ^-CTTTGTTACCATCGGTACATTCTT-3^- ^
**Secondary PCR**		
**MSP-1**	M1-KF	5^- ^-AAATGAAGAAGAAATTACTACAAAAGGTGC-3^- ^
	M1-KR	5^- ^-GCTTGCATCAGCTGGAGGGCTTGCACCAGA-3^- ^
	M1-MF	5^- ^-AAATGAAGGAACAAGTGGAACAGCTGTTAC-3^- ^
	M1-MR	5^- ^-ATCTGAAGGATTTGTACGTCTTGAATTACC-3^- ^
	M1-RF	5^- ^-TAAAGGATGGAGCAAATACTCAAGTTGTTG-3^- ^
	M1-RR	5^- ^-CATCTGAAGGATTTGCAGCACCTGGAGATC-3^- ^
		
**MSP-2**	M2-FCF	5^- ^-AATACTAAGAGTGTAGGTGCARATGCTCCA-3^- ^
	M2-FCR	5^- ^-TTTTATTTGGTGCATTGCCAGAACTTGAAC-3^- ^
	M2-ICF	5^- ^-AGAAGTATGGCAGAAAGTAAkCCTYCTACT-3^- ^
	M2-ICR	5^- ^-GATTGTAATTCGGGGGATTCAGTTTGTTCG-3^- ^

### Detection of Alleles

The secondary PCR products were separated by electrophoresis on 7% polyacrylamide gel in 1X TBE (Tris-borate EDTA) buffer stained with ethidium bromide. Bands were visualized by the gel document system (Infinity 3026, Vilber Lourmat, Marnela Valled, France) and the sizes of the fragments were estimated by comparison to the 100 bp DNA ladder (iNtRON Biotechnology, Inc. Seoul, Korea). Fragments representing the different alleles were selected and purified using a PCR purification kit (iNtRON Biotechnology, MEGAquick-spin PCR & Agarose Gel DNA Extraction System, Cat. no. 17281, Seoul, Korea). Purified PCR products for a limited number of isolates representing different alleles of *MSP-1 *and *MSP-2 *were sequenced in both directions with the primers of the secondary PCR using the ABI PRISM^® ^BigDyeTM terminator v3.0 Ready Reaction Cycle Sequencing Kit according to the manufacurer's instruction (Applied Biosystems, USA) in 3130 × l Genetic Analyzer (Applied Biosystems, USA). The sequences were used to correct the estimated molecular weight and to confirm the nature of the amplified product.

### Multiplicity of infection and heterozygosity

The multiplicity of infection (MOI) or number of genotypes per infection was calculated by dividing the total number of fragments detected in *MSP-1 *or *MSP-2 *by the number of samples positive for the same marker. Heterozygosity which represents the probability of being infected by two parasites with different alleles at a given locus and ranging between 0 and 1 was calculated by using the following formula: H_E _= [n/(n-1)] [(1-∑pi^2 ^)], where n is the number of isolates sampled and pi is the allele frequency at a given locus [5,9]. Isolates with more than one genotype were considered as polyclonal infection while the presence of a single allele was considered as monoclonal infection.

### Statistical analysis

Data was analyzed using the SPSS for windows software version 13. For descriptive analysis, proportion was used to present the distribution of different allelic families while the mean was used to present the MOI. Independent t-test was used to compare the mean MOI according to gender, time of the year of infection and parasitaemia. A *P *value of ≤ 0.05 was considered indicative of a statistically significant difference.

### Ethical consideration

The protocol of this study (Reference Number: 788.73) has been approved by the Medical Ethics Committee of the University of Malaya Medical Centre, Kuala Lumpur, Malaysia. The study was also registered with the National Medical Research Registry, Malaysia (Research ID: 5681). After a clear explanation of the objectives of the study, the subjects concerned agreed to participate in the study and verbal consents were obtained.

## Results

*P. falciparum *parasites were detected in a total of 75 samples. This includes 64 archived positive slides, and eleven blood samples out of 728 collected in the survey. Of the total positive samples, 66% were from males and 34% were from females with a mean age of 27.8 years and standard deviation of 4.1 years. Moreover, 64.2% of the positive samples collected were from Malay, 23.9% from aborigines, 6.5% from Indians and 2.7% from Chinese. In addition, two cases were detected from foreign workers who were residing in Malaysia for the past three years. The asexual *P. falciparum *parasitaemia from the collected samples ranged from 50 to 17,100 parasites/μl of blood with a geometric mean of 4,065 parasites/μl. Low parasitaemic individuals (parasite count < 1000 parasites/μl of blood) represented 41.3%, while moderate-to-high parasitaemia (parasite count ≥ 1000 parasites/μl of blood) were 58.7**%**.

### Multiplicity of infection and genetic diversity

All samples positive for *P. falciparum *were genotyped using *MSP-1 *(Block-2) and *MSP-2 *(Block-3) by nested PCR. Of these, 65 (87%) were found positive for *MSP-1 *and *MSP-2 *(40 with *MSP-1*only and 25 with both). Multiplicity of infection (MOI) was found to be 1.37 and 1.20 for *MSP-1 *and *MSP-2*, respectively (Table 2). Mean MOI was compared according to parasitaemia (low and moderate-to-high), year of infection (2007/2008 and 2009/2010) and sex of patients. The mean MOI was found to be higher in female patients, those diagnosed in year 2009/2010, and in individuals with moderate-to-high parasitaemia than male patients, those diagnosed in year 2007/2008, and in those with low parasitaemia. However, the differences in MOI between these groups were not statistically significant (P > 0.05).

**Table 2:** Multiplicity of infection, heterozygosity and type of infection for *MSP-1 *and *MSP-2 *genes of *P. falciparum *isolates from Pahang, Malaysia

Gene	MOI Mean (± SEM)	Heterozygosity	Monoclonal infection n (%)	Polyclonal infection n (%)
**MSP-1**	1.37 (± 0.1)	0.57	43 (66.2)	22 (33.8)

**MSP-2**	1.20 (± 0.09)	0.55	20 (80.0)	5 (20.0)

n: number of cases. SEM: standard error of mean

**Figure 2:**
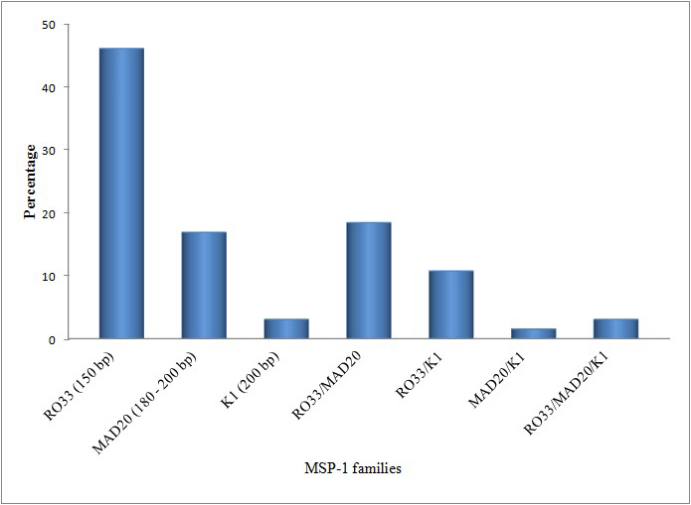
**Distribution of different allelic families of *MSP-1 *(n = 65)**.

**Figure 3:**
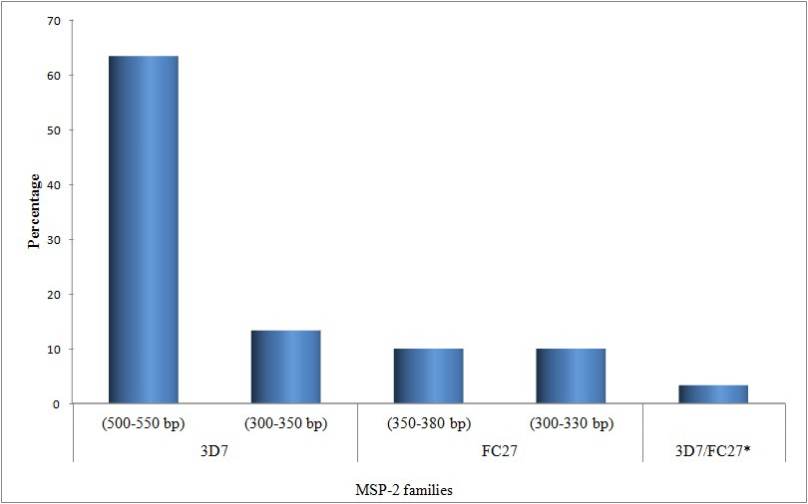
**Distribution of different allelic families of *MSP-2 *(n = 25)**. * 3D7 (300-350 bp)/FC27 (350-380 bp).

In *MSP-1*, RO33, MAD20 and K1 allelic families were identified with an overall frequency of 89. RO33 allelic family was predominant as it was identified in 80.0% (52/65) of the samples. The distribution of the identified *MSP-1 *allelic families is illustrated in Figure 2. One third (33.8%) of the blood samples positive for *MSP-1 *were identified as polyclonal infections while two-thirds (66.2%) were monoclonal infections. Among the polyclonal infections, RO33/MAD20 and RO33/K1 constituted 18.5% (12/65) and 10.8% (7/65), respectively. Trimorphic infections of *MSP-1*, ie. those having three allelic families, were also reported in 3.1% of the cases. With respect to *MSP-2*, 3D7 and FC27 allele types were detected among the isolates (Figure 3). The frequency of samples with only 3D7 allelic family [76.0% (19/25)] was found to be higher than the samples with only FC27 family [20.0% (5/25)]. However, *MSP-2 *with both allelic types was identified only in one sample (4.0%). On the other hand, monoclonal infection was reported in 80.0% (20/25) of cases positive for *MSP-2 *alleles while 20% were polyclonal.

### Allelic frequency and heterozygosity

The total number of detected alleles was seven. *MSP-1 *had three alleles; RO33 (150 bp), MAD20 (180 - 200 bp) and K1 (200 bp). Within *MSP-2 *block, 3D7 family had two different alleles; 3D7 (330 - 350 bp) and 3D7 (500 - 550 bp). Similarly, two different alleles were detected for FC27 family; FC27 (300 - 330 bp) and FC27 (350 - 380 bp). The frequencies of these alleles based on PCR products size (base pairs, bp) and their combinations are shown in Figures 2 and 3. Expected heterozygosity was almost the same for *MSP-1 *locus (H_E _= 0.57) and *MSP-2 *locus (H_E _= 0.55).

## Discussion

The present study is the first to provide information about the genetic diversity of *P. falciparum *in Malaysia. Our findings showed a low multiplicity of infection (MOI) for both *MSP-1 *and *MSP-2 *reflecting the low intensity of malaria transmission in Peninsular Malaysia. This is in agreement with previous observations of an increased MOI with increasing endemicity [10,11].

The present study showed that the frequency of the *MSP-1 *allelic families was higher than *MSP-2*. The predominant families in the *MSP-1 *and *MSP-2 *were RO33 and 3D7, respectively. These findings are in agreement with previous studies in Brazil and Gabon which demonstrated the predominance of the RO33 allelic family [12,13]. By contrast, previous studies in Thailand and Myanmar showed that MAD20 was the predominant allelic family while RO33 family showed either a very low frequency or not detected at all [8,14]. A topographic-based variation in the distribution of *MSP-1 *allelic families within the same area was described by a recent study from Indonesia which aimed at comparing the distribution of *MSP-1 *allelic families in coastal and mountainous areas in West Sumatera. This study reported that K1 and MAD20 were the most predominant allelic families in the coastal and mountainous areas, respectively [15].

The association between the distribution of allelic families and the severity of malaria has been investigated, and the studies have yielded a variety of results. RO33 allelic family was frequently reported in asymptomatic malaria cases and K1 family in severe cases [13,16]. This is supported by a community-based study in Papua New Guinea which reported an association between reduced risk of clinical malaria and infection with parasites of *MSP-1 *type RO33 or *MSP-2 *type 3D7 [17]. Moreover, a study from Senegal showed that *MSP-2 *was found more prevalent among patients with severe malaria than *MSP-1 *[18]. On the other hand, previous studies revealed an association between the dominance of K1 allelic family and the existence of asymptomatic malaria infection [19,20]. In this present study, we could not examine such association as the majority of samples were archived stained slides with unclear information on the severity of malaria infections. However, among the eleven positive blood samples collected by the survey, only one severe malaria case was reported. A previous community-based study which has been carried out in Pahang, Malaysia indicated that most of the malaria patients diagnosed during mass screening surveys conducted by malaria control units, were apparently healthy [21].

The present study found that about two-thirds of the *MSP-1 *positive cases harbored a single allele (monoclonal infection), and the polyclonal infection was reported in 33.8%. On the other hand, 80.0% of the *MSP-2 *positive cases had monoclonal infection. These findings suggested a low complexity of *P. falciparum *population. Polyclonal type infections were found in the mesoendemic areas up to 50% as against 100% in holoendemic areas [20,22,23]. Moreover, a previous study from Congo showed a significant association between the complexity of infection and polyclonal infections with the asymptomatic malaria [24]. Another study aimed at comparing the genetic diversity (based on *MSP-1*) of malaria parasite in different countries of different endemicity levels showed that the presence of polyclonal infection was more common in areas with high endemicity [25].

The present study reported few numbers of alleles (seven alleles for both *MSP-1 *and *MSP-2*) circulating in the study area with RO33 (150 bp) and 3D7 (500 - 550 bp) being highly frequent. We found that *MSP-2 *allelic families 3D7 and FC27 exhibit size polymorphism while *MSP-1 *allelic families K1, MAD20 and RO33 showed monomorphic pattern. Previous studies reported that *MSP-2 *gene is highly polymorphic compared to *MSP-1 *and among the *MSP-1 *alleles, RO33 found to be monomorphic compared to the polymorphic families K1 and MAD20 [8,26]. Moreover, a previous study from a low endemic country identified only one MAD20 allele of *MSP-1 *while RO33 or K1 alleles were not found in any sample [27]. It is reported that genomic DNA extracted from archived stained blood slides showed a relatively poor performance at low level parasitaemia and this may explain the low detection of *MSP-2 *[28]. However, we should indicate that all blood samples collected by the survey were found to be positive for both *MSP-1 *and *MSP-2*.

The low allelic diversity together with the high frequency of the circulating alleles increase the chance of the re-infection with the same allele, decreasing the discriminatory power of *MSP-1 *and *MSP-2 *to differentiate between recrudescence and re-infection in Peninsular Malaysia. Thus, the genotyping of *P. falciparum *based on *MSP-1 *and *MSP-2 *in antimalarial drug efficacy trials, in Peninsular Malaysia, may lead to misclassification of re-infection as recrudescence (treatment failure) and cause unnecessary change of malaria drug policy. However, the possibility of re-infection during the follow-up may be low in an area with low intensity of transmission. In the same vein, heterozygosity was 0.55-0.57 suggesting that the parasite population in Pahang, Malaysia exhibits intermediate heterozygosity, which is consistent with the heterozygosity range (0.51-0.65) reported in Southeast Asia/Pacific region [29]. In areas with declining endemicity, it is reported that the number and diversity of alleles (heterozygosity) decrease with decreasing *P. falciparum *transmission [29,30].

The correlation between the genetic variation of *P. falciparum *and malaria endemicity has been described [10,11]. Our findings showed no significant difference in the mean MOI according to sex of patients, level of parasitaemia, and years of infection. This is in agreement with previous reports in other countries [31,32]. In contrast, previous studies from Senegal, Mozambique and Iran showed a significantly high MOI in patients with moderate-to-high parasitaemia [33,34]. In the present study, most of the positive samples were from adult patients aged 22-40 years, and this limited range of age constraint examining the difference in MOI according to age. However, previous studies showed a pattern of greater MOI in older individuals than younger individuals reflecting more previous exposure to infection [35,36]. Conflicting findings, indicated decreased MOI with age, were also reported [32,37].

## Conclusions

This study provides information on the genetic diversity of *P. falciparum *in Malaysia based of *MSP-1 *and *MSP-2*. Malaria due to *P. falciparum *in Pahang state of Malaysia was found mostly to be monoclonal infection with generally low parasite diversity, together with high predominance of RO33 allelic family of *MSP-1 *and 3D7 family of *MSP-2*. The low allelic diversity with the high frequency of alleles may limit the use of MSP-based genotyping in antimalarial drug efficacy trials in Peninsular Malaysia.

## Competing interests

The authors declare that they have no competing interests.

## Authors' contributions

WMA, HMA, MAKM and JS designed the study; WMA and AMA the field study, carried out the laboratory work and collated the data; WMA and HMA performed the statistical analysis; MAKM and RSA provided technical advisory support in genotyping and data interpretation; WMA, HMA and MAKM drafted the primary version of the manuscript; JS and RSA contributed to the revision of the manuscript. All authors read and approved the final manuscript.
